# Towards Room Temperature Phase Transition of W-Doped VO_2_ Thin Films Deposited by Pulsed Laser Deposition: Thermochromic, Surface, and Structural Analysis

**DOI:** 10.3390/ma16010461

**Published:** 2023-01-03

**Authors:** Yannick Bleu, Florent Bourquard, Vincent Barnier, Anne-Sophie Loir, Florence Garrelie, Christophe Donnet

**Affiliations:** 1 Université de Lyon, Université Jean Monnet-Saint-Étienne, CNRS, Institut d’Optique Graduate School, Laboratoire Hubert Curien, UMR 5516, F-42023 Saint-Etienne, France; 2 Mines Saint-Etienne, Université de Lyon, CNRS, UMR 5307 LGF, Centre SMS, F-42023 Saint-Etienne, France

**Keywords:** vanadium dioxide, W doping, phase transition temperature, luminous transmittance, ARXPS

## Abstract

Vanadium dioxide (VO_2_) with an insulator-to-metal (IMT) transition (∼68 °C) is considered a very attractive thermochromic material for smart window applications. Indeed, tailoring and understanding the thermochromic and surface properties at lower temperatures can enable room-temperature applications. The effect of W doping on the thermochromic, surface, and nanostructure properties of VO_2_ thin film was investigated in the present proof. W-doped VO_2_ thin films with different W contents were deposited by pulsed laser deposition (PLD) using V/W (+O_2_) and V_2_O_5_/W multilayers. Rapid thermal annealing at 400–450 °C under oxygen flow was performed to crystallize the as-deposited films. The thermochromic, surface chemistry, structural, and morphological properties of the thin films obtained were investigated. The results showed that the V^5+^ was more surface sensitive and W distribution was homogeneous in all samples. Moreover, the V_2_O_5_ acted as a W diffusion barrier during the annealing stage, whereas the V+O_2_ environment favored W surface diffusion. The phase transition temperature gradually decreased with increasing W content with a high efficiency of −26 °C per at. % W. For the highest doping concentration of 1.7 at. %, VO_2_ showed room-temperature transition (26 °C) with high luminous transmittance (62%), indicating great potential for optical applications.

## 1. Introduction

VO_2_ has the closest phase transition to room temperature of any the thermochromic oxide materials and has consequently been extensively studied for a variety of applications including electronic switches, smart windows, memory devices, RF microwave switches, and terahertz metamaterial devices [1,2,3,4,5]. Indeed, VO_2_ enables insulator-to-metal transition (IMT) as well as transition from a monoclinic (M1) to a rutile structure (R) at around 68 °C [6]. However, the phase transition temperature of pristine VO_2_ thin films is too high for room temperature applications. To meet the demand for a broad range of room temperature applications, considerable efforts have been made to reduce the phase transition temperature. One way to do so is replacing V^4+^ ions by metal ions with higher valences such as W^6+^, Nb^5+^, and Mo^6+^ [7,8,9,10], among which W has been considered one of the most effective dopants. Interestingly, the doping of W into the VO_2_ matrix affects the transition characteristics. The substitutional doping of the W^6+^ to replace V^4+^ has been shown to lead to a remarkable reduction in the phase transition temperature, i.e., 20–28 °C per W at. % [7,10,11,12,13,14]. VO_2_-based thin films can be synthesized using several techniques including sol-gel [15], magnetron sputtering [16,17], chemical vapor deposition [18], and pulsed laser deposition (PLD). However, there have been few reports on synthesizing W-doped VO_2_ films using PLD [19,20,21,22], which is known to be a versatile method to control doping in thin films. Komal et al. [23] used mixed V_2_O_3_ and WO_3_ pellets as targets for the PLD process and observed that the VO_2_ doped with 1.5 at. % of W reduced the phase transition temperature towards room temperature (27 °C). Émond et al. [24] used PLD to prepare W-doped VO_2_ films with a phase transition temperature of 36 °C. Soltani et al. [25] manufactured 1.6 at. % W-doped VO_2_ thin films using a W-doped vanadium oxide target. The phase transition temperature was about 36 °C and all-optical switching was demonstrated at a telecommunication wavelength of 1.55 µm. S. S. Majid et al. [26] used a W-doped target and obtained W-doped VO_2_ films with a significantly lower phase transition temperature (∼19 °C for 1.3% W) by stabilizing the metallic rutile phase R. All these studies demonstrate that PLD is a versatile method capable of tuning the properties of VO_2_. However, they used either a target mixture of tungsten and vanadium oxide or a co-ablation process and mainly focused on reducing the phase transition temperature. In the present work, an innovative method to synthesize W-doped VO_2_ thin films using pulsed laser deposition is proposed. This method consists in fabricating multilayers composed of V/W (+O_2_) and V_2_O_5_/W bilayers, followed by rapid thermal annealing to allow both crystallization of VO_2_ and the diffusion of W through the VO_2_ layers to synthetize homogeneous W-doped VO_2_ thin films. The main advantage of this process over co-ablation deposition is its repeatability. Indeed, co-ablation can cause problems of thickness and of non-homogeneous composition as well as contamination between the targets. In addition to the synthesis process, this work not only aims to reduce the phase transition temperature but also to improve our understanding of the surface chemistry of W-doped films. For the latter, angle-resolved X-ray photoelectron spectroscopy (ARXPS), which is rarely used in VO_2_-reported studies, was used to measure the concentration of vanadium, the depth of tungsten oxidation, as well as to assess their depth distribution.

## 2. Materials and Methods

### 2.1. Synthesis of W-Doped VO_2_ Thin Films Using Pulsed Laser Deposition

W-doped VO_2_ thin films were synthesized by rapid thermal annealing of multilayers composed of VO_X_/W bilayers as illustrated in the figure inserted in Table 1. The bilayers were deposited on fused silica substrates using pulsed laser deposition (PLD) at room temperature. The PLD chamber was evacuated to a background pressure of 2 × 10^−7^ mbar. Three types of targets were used in the investigation: Vanadium (V), Vanadium pentoxide (V_2_O_5_), and Tungsten (W), all at 99.9% purity. A KrF excimer laser (wavelength 248 nm, repetition rate 10 Hz, fluence 13 J/cm^2^) was used to ablate the targets. The target/substrate distance was 5 cm. The laser beam was oriented with an angle of 45° to the target. For the W-doped VO_X_ using metallic vanadium as a precursor, the oxygen pressure inside the deposition chamber was 1 × 10^−2^ mbar when ultrapure O_2_ was used. For the W-doped VO_X_ using the V_2_O_5_ target, ablation was performed without oxygen gas. During the deposition process, the targets were rotated to enable uniform ablation. For all depositions, the deposition rate obtained by profilometry was around 6.3 nm/min for V+O_2_, 6.4 nm/min for V_2_O_5_, and 1.37 nm/min for W. The alternative ablation sequences of V2O5 (or V in O_2_) and W were adjusted to obtain three multilayers (A, B, C) with different W contents to obtain three W-doped VO_X_ thin films with a similar total thickness of 20 nm (Table 1).

The rapid thermal annealing process was performed using an RTP furnace (AS-One RTP system from Annealsys, Montpellier, France). For samples A and B, the annealing temperature was 400 °C for 120 s, while for sample C, the annealing temperature was 450 °C for 60 s. The time of rapid thermal annealing was adjusted from preliminary experiments, depending on the two different precursors, to ensure the observation of the thermochromic transition in both cases. All the annealing processes were carried out at an oxygen partial pressure of 1 mbar and a 50 sccm flow. The heating rate was 5 °C/s and the cooling was natural. The annealing chamber was evacuated to a 1 × 10^−2^ mbar before the oxygen was introduced. Undoped VO_2_ thin films were obtained using a similar process for the purpose of comparison.

### 2.2. Characterization of the Undoped VO_2_ and W-Doped VO_2_ Films

The composition and dopant profile were investigated using XPS analysis using a Thermo VG Theta probe spectrometer (Thermo Fisher Scientific, Invitrogen, Waltham, MA, USA) with a focused monochromatic AlKα source (hν = 1486.68 eV, 400 μm spot size) and photoelectrons were collected using a concentric hemispherical analyzer. A constant ΔE mode and a 2D channel plate detector (Thermo Fisher Scientific, Invitrogen, Waltham, MA, USA) were used. The energy scale was calibrated with sputter-cleaned pure reference samples of Au, Ag, and Cu such that Au4f_7/2_, Ag3d_5/2_, and Cu3p_3/2_ were positioned at binding energies of 83.98, 386.26, and 932.67 eV, respectively. Angle-resolved XPS analyses were performed thanks to the ability of the spectrometer to simultaneously collect several photoelectron emission angles in the acceptance range of 60° without tilting the sample. Charge neutralization was applied during analysis. High-resolution spectra (i.e., O1s-V2p, V3p-W4f) were fitted using AVANTAGE software version 5.9 by Thermo Fisher Scientific. The structure of the films was analyzed by Raman analysis (Jobin-Yvon ARAMIS) using a Helium-Neon laser source (Horiba Jobin Yvon, Gières, France) at an excitation wavelength of 633 nm, a laser power of 0.1 mW, focused with a 100× objective, consistent with a spot diameter of less than 1 mm. Atomic force microscopy (AFM) (Icon BRUKER, Berlin, Germany) and scanning electron microscopy (SEM) (JEOL IT 800 SHL, Tokyo, Japan) were used to analyze the topography, roughness, and morphology of the films. The thermochromic properties of the films were measured by collecting the transmittance in a temperature range between 15 and 100 °C using a fiber optic spectrometer (Ocean Insight, Duiven, The Netherlands) equipped with handmade heating units in the visible (400–800 nm) and IR (900–2500 nm) wavelength ranges. For the undoped and W-doped VO_2_ thin films, the integrated luminous transmittance (T_lum_, 380–780 nm) and solar modulation efficiency (T_sol_, 300–2500 nm) were deduced from the following equation:

T_lum, sol_ = (∫φ_lum, sol_ (λ)T(λ)dλ)/(∫φ_lum, sol_ (λ)dλ) (1)

where T(λ) is film transmittance at wavelength (λ), φ_lum_(λ) is the standard luminous efficiency depending on the photopic vision of human eye, φ_sol_(λ) is the solar irradiance spectrum (air mass 1.5) corresponding to the sun at an angle of 37° to the horizon [23]. ΔT_sol_ is obtained from ΔT_sol_ = T_sol_ (15 °C) − T_sol_ (100 °C) and T_lum_ = (T_lum_ (15 °C) + T_lum_ (100 °C)/2).

## 3. Results and Discussion

### 3.1. Thermochromic Properties

To investigate the influence of W-doping on thermochromic properties, the transmittance of the samples during a heating and cooling phase were recorded and plotted the hysteresis loops of undoped and W-doped thin films.

**Figure 1:**
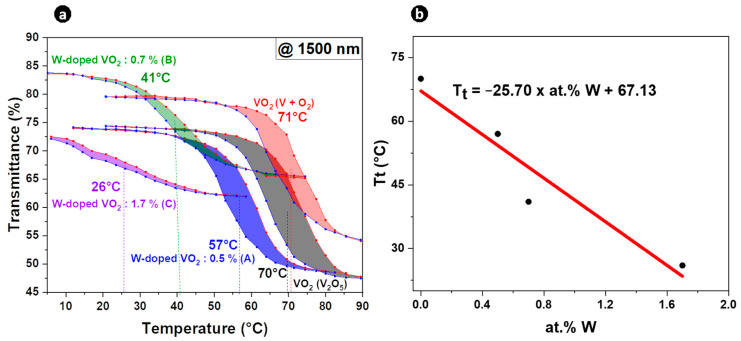
(**a**) Hysteresis loops derived from the temperature-dependent transmittance of the undoped and W-doped (A, B, and C) thin films at a wavelength of 1500 nm. (**b**) The correlation between the phase transition temperature T_t_ and W concentration.

Figure 1a shows the hysteresis loop produced by plotting the temperature dependence of transmittance of undoped and W-doped VO_2_ thin films at a fixed wavelength of 1500 nm. The switching temperatures during heating (T_t,h_) and cooling (T_t,c_) were determined from the half-value width of each curve and the average temperature of commutation (T_t_) representative of thermochromic behavior was defined as T_t_ = (T_t,h_ + T_t,c_)/2. Both undoped VO_2_ films had a phase transition temperature Tt of 70–71 °C (quite similar whatever the precursors). 

The dependence of the phase transition temperature on the tungsten content was linear (Figure 1b). W doping significantly reduced T_t_ to ~−26 °C per at. % W, as shown in Figure 1b, which is in line with reported values of ~20–28 °C per at. % W [7,12,13,14]. The T_t_ reduction mechanism of the W-doped VO_2_ can be ascribed to free electron carriers generation [27] as well as to the extra strain resulting from the atom replacement of V^4+^ by W^6+^. This induced high symmetry around the W atom, implying transformation into a rutile structure [28,29], which was subsequently corroborated by Raman analysis.

**Figure 2:**
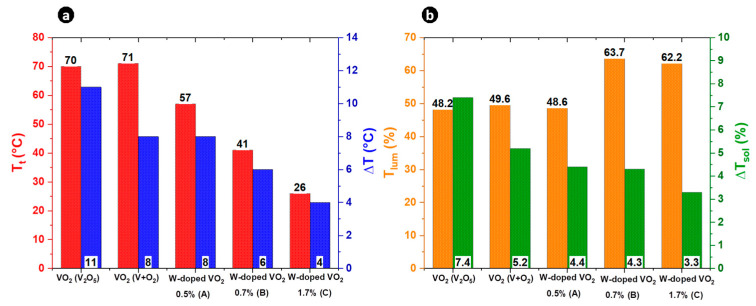
(**a**) Phase transition temperature (red) and hysteresis width (blue) for undoped and W-doped VO_2_ thin films. (**b**) The corresponding luminous transmittance (T_lum_, orange) and the solar modulation ability (ΔT_sol_, in green) values.

Hysteresis behavior is another important thermochromic parameter of VO_2_-based thin films. The hysteresis loop width gradually narrowed from 11 °C for the VO_2_ film to 4 °C for the film doped with 1.7 at. % W, as can be seen in Figure 2a. This illustrates that the W dopant not only reduces the transition temperature but also reduces the width of the hysteresis loop. Previous studies suggested that ΔT is closely linked to the grain size, lattice stress, impurity phase, and crystallinity of VO_2_ film [30,31]. Structural defects induced by W doping act as nucleation sites of the phase transition [32,33]. Therefore, the activation energy of the phase transition would be reduced, with a decrease in hysteresis width. The result is promising, as narrow hysteresis loops are required to create devices with rapid commutations.

Moreover, the luminous transmittance (T_lum_) and the solar modulation ability (ΔT_sol_) are plotted in Figure 2b. Compared to undoped VO_2_ thin films, the luminous transmittance of the W-doped VO_2_ thin films was higher. Indeed, T_lum_ increased with increasing W content. The luminous transmittance of the W-rich sample was 62.2%, a rather very good performance for W-doped VO_2_ thin films. In the literature, the luminous transmittance of VO_2_ after W-doping usually either remained unchanged or decreased, as reported in Table 2. In the present study, our W-doped VO_2_ thin films had higher T_lum_. Further investigations would be necessary to identify the exact origin of such a significant increase of T_lum_ at the two highest W contents. The solar modulation ability ΔT_sol_ decreased gradually with increasing W content. This is consistent with the results of previous works [34,35], in which ΔT_sol_ decreased with increasing W content. The variation of ΔT_sol_ depending on W content is in good agreement with the variation of T_t_. Two explanations are possible: the incorporation of W induces a destabilization of VO_2_ (M) lattice, and reduces the phase transition temperature. On the other hand, too much W doping leads to an excess of free electrons, thereby deteriorating the phase transition property [36]. Overall, as shown in Table 2, our W-doped VO_2_ thin films outperformed other reported works on W-doped VO_2_ using different synthesis methods in terms of reducing T_t_ and improving luminous transmittance.

### 3.2. Surface Chemistry Analysis: Composition and Depth Distribution

**Figure 3:**
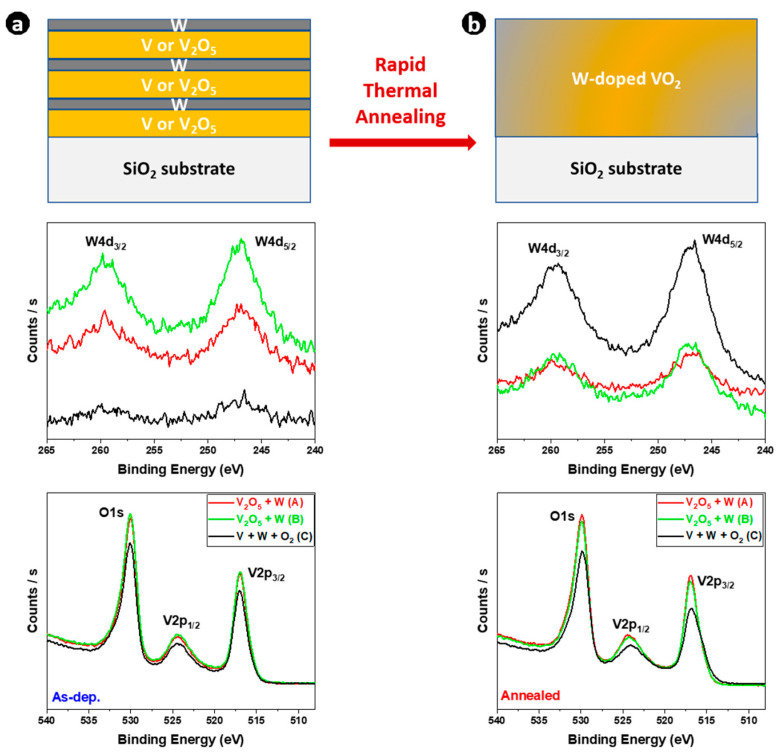
V 2p and W 4d core-level XPS spectra of (**a**) W-doped VO_X_ as-deposited thin films, and (**b**) W-doped VO_2_ thin films after rapid thermal annealing.

To analyze the surface chemistry, the high-resolution XPS spectra of V 2p and W 4d core-levels of W-doped VO_X_ (as-deposited) and W-doped VO_2_ (after annealing) thin films are shown in Figure 3a,b, respectively.

**Figure 4:**
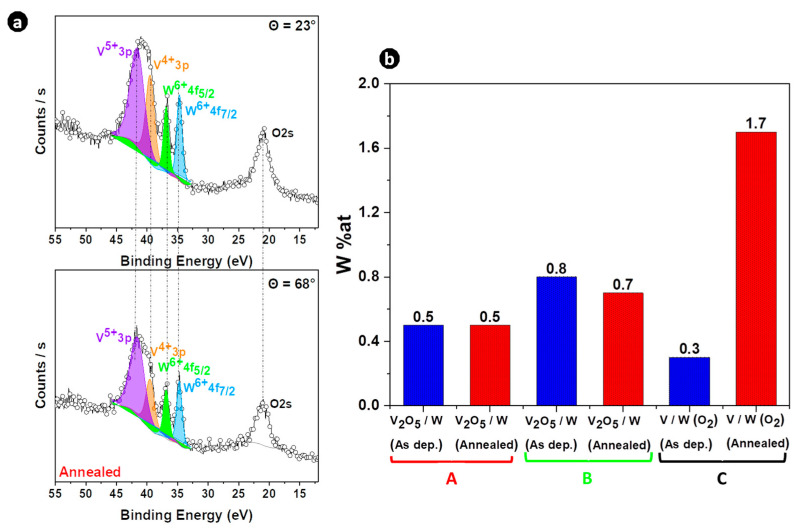
(**a**) Fitted ARXPS spectra of V 3p, W 4f, and O 2s core-levels for sample C after thermal annealing, at detection angles of 23° and 68°. (**b**) Quantification of W based on O 2s, W 4f, and V 3p peaks of the different samples before and after rapid thermal annealing.

The W peaks of W 4d_5/2_ and W 4d_3/2_ were located at 246.8 and 259.8 eV, respectively, reflecting the + 6 oxidation state of W ions in both thin films [11,12,41,44]. The V 2p core-level was split into two regions, V 2p_3/2_ located at 516.9 eV and V 2p_1/2_ located around 524 eV [45,46], with the O1s located at 530 eV. The intensity of vanadium and oxygen peaks is not the same for all films, especially when the two deposition precursors V/W (O_2_) and V_2_O_5_/W are compared. Since the intensity of the tungsten peak differed from one sample to another, the W content differed. The presence of W can also be confirmed by its W 4f valence state [11]. The angle-resolved XPS spectra of W 4f core-levels at 23 and 68° of sample C are shown in Figure 2a after thermal annealing. Two strong peaks of W 4f core-level located at 34.7 eV and 36.9 eV were assigned to W 4f_7/2_ and W 4f_5/2_, respectively. This revealed that W^6+^ cations were present in W-doped VO_2_ thin films in line with previous reported work [47], and confirmed the successful W-doping of VO_2_ thin films. Furthermore, the atomic concentrations of W were calculated based on the XPS spectra of V 3p and W 4f and the results of the quantification are shown in Figure 4b.

Figure 4b reports W content in each sample before and after annealing. The annealing process did not significantly affect W content when the V_2_O_5_ target was used for W-doped VO_2_ synthesis (samples A and B) because almost identical W contents were found consecutive to the annealing treatment. This suggests that V_2_O_5_ may act as a barrier to the surface diffusion of W. On the contrary, using the vanadium target, an increase in the W content after thermal annealing (sample C) was observed. This means that W diffused more towards the VO_2_ surface during annealing and suggests that the V+O_2_ environment favors the surface diffusion of W. Such an increase in W content during annealing was also recently reported by Ström et al. [48]. In their study, the W content in steel increased during thermal annealing and the modified composition was attributed to a near-surface effect. This might also be the case in our study, with surface diffusion of W towards the VO_2_ surface during the annealing process.

**Figure 5:**
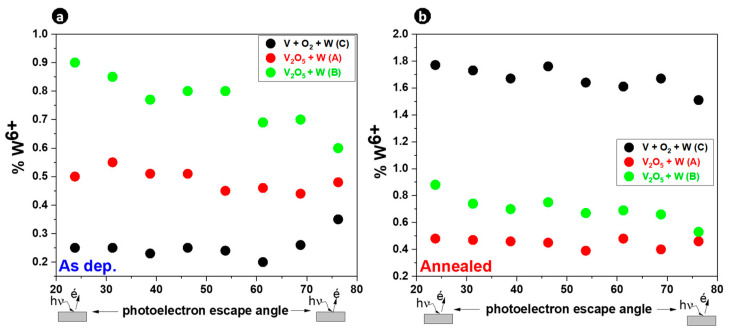
The proportion of W^6+^ based on V 3p, W 4f, and O 2s as a function of the ARXPS detection angle for all the samples (**a**) before thermal annealing and (**b**) after thermal annealing.

For more insight into the depth distribution of W, Figure 5a,b show the dependence of the W^6+^ proportion as a function of the detection angle (23 to 76°) for all the samples before and after thermal annealing.

Similar W distribution between as-deposited and annealed films in samples A and B showed that the inter-diffusion between VO_X_ and W layers occurred at room temperature during deposition. In other words, the inter-diffusion process and the crystallization process occurred separately. Diffusion occurred during PLD deposition while crystallization occurred during the thermal annealing stage. Conversely, with sample C, the different W distribution between as-deposited and annealed films showed that the inter-diffusion between VO_X_ and W layers was not completed during PLD deposition, but was completed during the thermal annealing stage. This surface diffusion leads to an increase in W content toward the surface after thermal annealing. Therefore, along the first nanometers probed by ARXPS, sample C was richer in W after thermal annealing, while samples A and B had lower W contents. In addition, W distribution was almost constant with few discrepancies irrespective of the sample (before and after annealing), meaning that the W distribution was homogeneous in all the samples.

**Figure 6:**
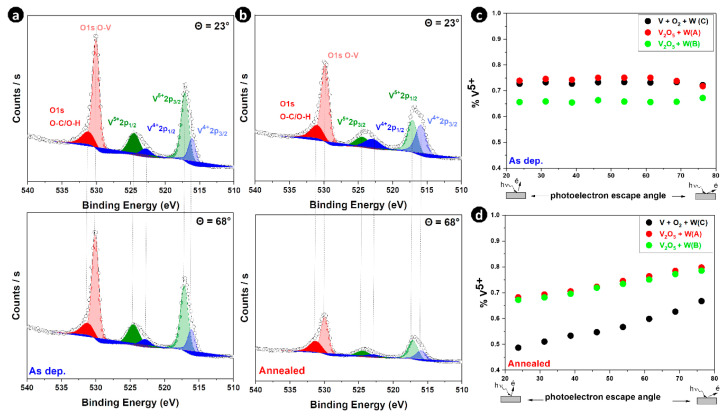
Fitted ARXPS spectra of V 2p and O 1s core-levels for sample C at detection angles of 23° and 68°, respectively, (**a**) before thermal annealing and (**b**) after thermal annealing. The concentration of the V^5+^ oxidation state of vanadium was obtained from the fitted spectra of the V 2p electrons as a function of the angle of detection in all the samples, (**c**) before thermal annealing and (**d**) after thermal annealing.

It is generally accepted that in addition to the doping element, the valence states of V can significantly affect the thermochromic characteristics of VO_2_-based thin films. Figure 6a,b show the high-resolution spectra of V 2p—O 1s recorded in XPS angle-resolved mode at two photoelectron take-off angles (23° and 68°) before and after thermal annealing for the W-rich sample (C). Deconvolution analysis of the V 2p_3/2_ and V 2p_1/2_ regions revealed two vanadium components in the samples, corresponding to V^4+^ and V^5+^ valence states: V^4+^ located at 516.1 eV for 2p_3/2_, and 522.8 eV for 2p_1/2_ with V^5+^ centered at 517.1 eV for 2p_3/2_, and 524.5 eV for 2p_1/2_ [45,46]. The binding energies of the peaks at 515.8 eV and 517.2 eV are close to those reported for VO_2_ (515.7–516.2 eV) and V_2_O_5_ (516.9–517.2 eV), respectively [45,49,50]. There was no significant shift in the position of the peak in terms of the binding energy of the as-deposited and annealed samples. However, no difference in the intensity of the two oxidation states V^4+^ and V^5+^ was observed: the intensity of the V^4+^ oxidation state increased while that of V^5+^ decreased after thermal annealing. The O 1s peak is deconvoluted into two peaks, one corresponding to the O-C/O-H bond, which appeared at 531.2 eV due to surface contamination of the films. The other peak was due to the O-V bond located at 529.9 eV [51].

To investigate the depth distribution of the V^5+^ oxidation state of vanadium, the spectra of V 2p were measured at various detection angles ranging from 23° to 76° using angle-resolved X-ray photoelectron spectroscopy. Figure 6c,d shows the dependence of the concentration of the V^5+^ oxidation state as a function of the detection angle for all the samples before and after thermal annealing. It can be seen from Figure 6c that the angle dependence of V^5+^ oxidation state concentration is quite similar to that before thermal annealing, indicating homogeneous distribution of V^5+^ on the surface of all samples. After thermal annealing (Figure 5d), the proportion of the V^5+^ oxidation state increased with the increase in the detection angle in all samples. The results of angle-resolved measurements confirmed that the outer part of the W-doped VO_2_ thin films was enriched in V^5+^, especially when the V_2_O_5_ target was used for the synthesis of W-doped VO_2_ films and hence, that the other V^4+^ oxidation state is located in the inner region of the films. To summarize, sample C was poorer in V^5+^ and richer in W while samples A and B were richer in V^5+^ and poorer in W after thermal annealing.

### 3.3. Structural and Morphological Analysis

The structural change that occurred with W doping at room temperature was investigated using Raman analysis.

**Figure 7:**
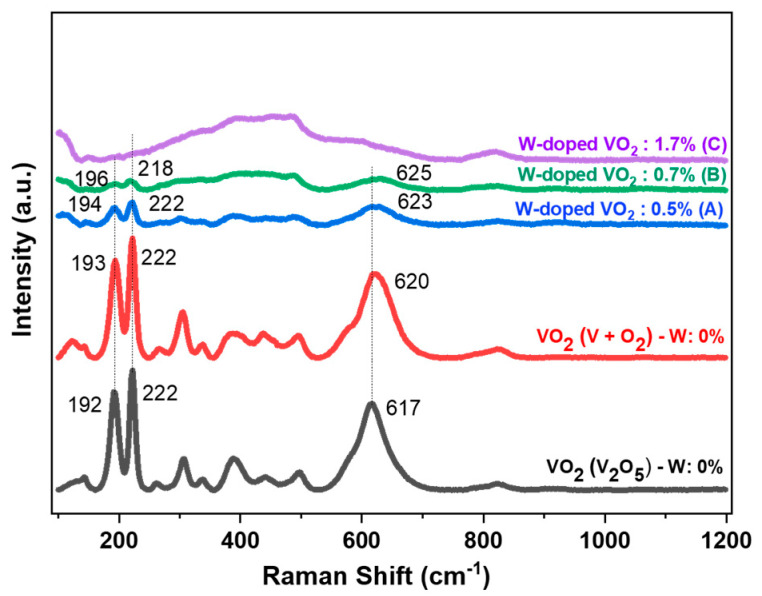
Raman spectra of undoped and W-doped VO_2_ (samples A, B, and C) thin films.

Figure 7 shows the Raman spectra of the undoped and W-doped thin films at room temperature. The two undoped VO_2_ films (black and red curves in Figure 7) had typical peaks at 143 (Ag), 192 (Ag), 222 (Ag), 262 (Bg), 306 (Ag), 338 (Ag), 389 (Ag), 441 (Bg), 498 (Ag), and 617 (Ag) cm^−1^, corresponding to the monoclinic VO_2_ (M1) phase [52,53,54]. No vibration peak of V_2_O_5_ was observed, perhaps due to the low concentration of V_2_O_5_ resulting in weaker molecular vibration. Concerning the W-doped VO_2_ films, a decrease in the intensities of the Raman active modes (low-frequency and high-frequency mode) and broadening of the peaks with an increase on the percentage of W doping were observed. This can be explained by the fact that W doping starts to favor a more symmetric rutile structure. A similar effect has already been reported for W-doped VO_2_ films [23,55] and Nb-doped VO_2_ films [56]. Moreover, the 617 (Ag) cm^−1^ phonon mode was slightly upshifted and broadened as well as being reduced in intensity with W doping up to 625 cm^−1^ (green curve), indicating a distorted M1 phase and the nucleation of rutile domains at this stage [57]. In the W-rich sample (1.7 at. % W), some modes started to disappear (purple curve), indicating the beginning of the metallic rutile phase as observed in the above-mentioned analysis of the phase transition temperature.

**Figure 8:**
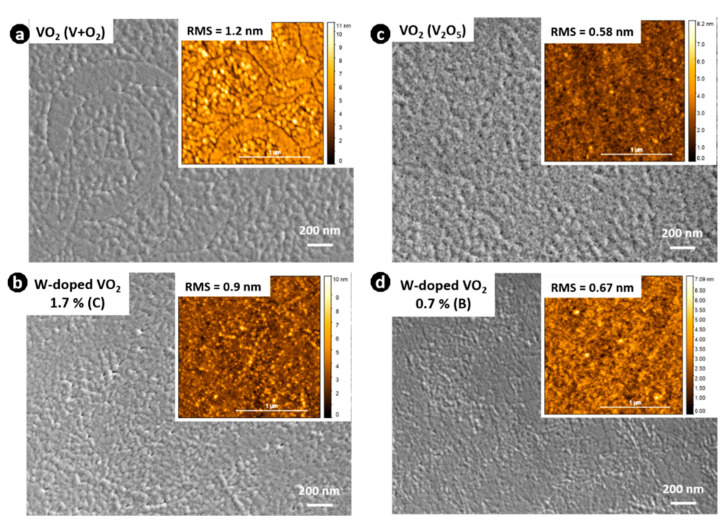
Top-view SEM micrographs of the obtained thin films after rapid thermal annealing (**a**) VO_2_ thin film obtained from V+O_2_. (**b**) W-doped VO_2_ (1.7 at. % W, sample C). (**c**) VO_2_ thin film obtained from V_2_O_5_. (**d**) W-doped VO_2_ thin film (0.7 at. % W, sample B). The inserts correspond to the AFM images.

Changes in the surface morphology and variations in the surface roughness of the undoped and W-doped VO_2_ thin films were investigated by SEM and AFM, respectively. Regarding the thin films obtained using V+O_2_ deposition, the surface morphology of undoped film (Figure 8a) was flat with trace-like cracks whereas with W doping, the surface of the film contained holes resembling pores. The surface morphology of the films deposited from the V_2_O_5_ target was uniform, dense, and compact, the undoped film being slightly smoother than the W-doped film. The difference in morphology between undoped and W-doped VO_2_ films can be attributed to the general regularity observed in solid solution formation: an increase in the number of constituents can play a role in the crystallization of the films [58,59]. AFM scanning maps of the films obtained from V+O_2_ exhibited a slight decrease in the root mean square (RMS) from the undoped to W-doped VO_2_ films. The RMS of the W-doped VO_2_ film obtained from V_2_O_5_, was slightly higher than that of the undoped film. However, the low RMS values indicate that our VO_2_-based thin films are extraordinarily smooth with improved quality compared to those obtained using other synthesis methods [60,61].

## 4. Conclusions

An innovative PLD method was developed to synthesize smooth W-doped thin films using V/W (+O_2_) and V_2_O_5_/W multilayers with subsequent post-rapid thermal annealing. Our results revealed that the inter-diffusion between the W and VO_X_ layers was completed during the PLD deposition process at room temperature when the V_2_O_5_ target was used, whereas when the V+O_2_ source was used, inter-diffusion between the W and VO_X_ layers was completed during the thermal annealing stage. Therefore, the V_2_O_5_ acted as a W diffusion barrier, while the V+O_2_ environment favored surface diffusion of W during the annealing stage. These findings also show that the V^5+^ is more surface sensitive and that the distribution of W on the surface of the samples is homogeneous. Furthermore, as expected, W doping leads to a sharp decrease in the VO_2_ IMT by a highly efficient reduction of the phase transition temperature −26 °C per at. % W. With W-doping of 1.7 at. %, the concentration of V^5+^ decreased, and the transition temperature of VO_2_ dropped to room temperature (26 °C), accompanied by a high luminous transmittance (62%) while retaining a narrow hysteresis width. These very good thermochromic properties have high potential in the application of smart windows and optical devices based on thermochromic switching behavior.

## Figures and Tables

**Table 1:** Condition of deposited multilayers using pulsed laser deposition. The ablated thickness per layer and the number of individual layers were adjusted to obtain three different W contents (next investigated using XPS).

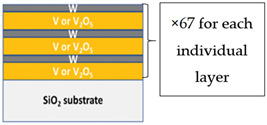	Sample A	Sample B	Sample C
V ablated thickness per layer (nm)	/	/	0.283
V_2_O_5_ ablated thickness per layer (nm)	0.283	0.270	/
W ablated thickness per layer (nm)	0.015	0.03	0.015
Number of VOx and W layers	67	67	67
Total thickness (nm)	20	20	20

**Table 2:** Comparison of some of the W-doped VO_2_ reported works on thermochromic parameters including T_t_, ΔT, T_lum_, and ΔT_sol_, with those of our present study. The terms « unchanged, increased, decreased » correspond to changes in the T_lum_ and ΔT_sol_ values of W-doped VO_2_ compared to those of undoped VO_2_.

W (at. %)	T_t_ (°C)	∆T (°C)	T_lum_	∆T_sol_	Process	Structural Form	Ref.
0.14	64	6.8	Unchanged	Decreased	Electron beam evaporation	Thin film	[11]
2	28	15	Unchanged	Decreased	Spin coating	Thin film	[37]
0.4	43	/	Increased	Decreased	Hydrothermal	Mesoporous film	[38]
0.8	36	20.5	Decreased	Decreased	Hydrothermal	Nanoparticles	[39]
3	44.4	18.4	Increased	Increased	Spin coating	Coating	[40]
2	43	/	Decreased	Decreased	Microemulsion technology	Nanostructure	[41]
1.3	55	/	Decreased	Decreased	Magnetron sputtering	Thin film	[42]
1.3	17.5	25.1	Increased	Decreased	Hydrothermal	Nanoparticle	[43]
0.7	41	6	Increased	Decreased	PLD	Thin film	This work
1.7	26	4	Increased	Decreased	PLD	Thin film	This work

## Data Availability

Data is available from the corresponding author upon reasonable request.
